# A Probabilistic Framework for Multimedia Dioxin Risk Assessment in Susceptible Populations

**DOI:** 10.3390/toxics14070571

**Published:** 2026-06-29

**Authors:** Kuan-Yi Chen, Chien-Cheng Jung, Ken-Hui Chang, Chow-Feng Chiang

**Affiliations:** 1 Department of Public Health, China Medical University, Taichung 406040, Taiwan; u105050002@cmu.edu.tw (K.-Y.C.); ccjung@mail.cmu.edu.tw (C.-C.J.); 2 Department of Safety Health and Environmental Engineering, National Yunlin University of Science and Technology, Yunlin 640301, Taiwan; ken@airlab.yuntech.edu.tw

**Keywords:** Monte Carlo simulation, AERMOD, MEPAS, age-dependent adjustment factors, municipal solid waste incineration

## Abstract

In this study, a probabilistic framework was developed to address uncertainty in multimedia risk assessment for susceptible populations while capturing population variability, illustrated by dioxin emissions from municipal solid waste incinerators. The framework integrates emission estimation, AERMOD dispersion modeling, and MEPAS multimedia modeling. Cancer risks were evaluated with dose-based slope factors and age-dependent adjustment factors (ADAFs) for early-life susceptibility. Three assessment approaches were compared: deterministic without ADAF adjustment, deterministic with ADAF adjustment, and probabilistic with ADAF adjustment. For the general population (*n* = 7058), stratified into adults and two susceptible subgroups, the P95 risks across all simulated grids (*n* = 139,287) increased from 5.5 × 10^−9^ to 1.4 × 10^−8^ (2.54-fold) after ADAF adjustment and further to 6.6 × 10^−8^ (an additional 4.71-fold) when inter-individual variability in IR/BW and CR/BW was incorporated through bivariate Monte Carlo simulation. For school children (*n* = 1879), the corresponding P95 risks increased from 6.9 × 10^−10^ to 3.0 × 10^−9^ (4.35-fold) and then to 6.3 × 10^−9^ (an additional 2.1-fold). The stepwise increase in risk across these approaches illustrates how the proposed framework reduces uncertainty and quantifies variability. Additional analysis examined uncertainty associated with gas–particle partitioning, ADAF adjustment, and inhalation slope factor extrapolation.

## 1. Introduction

Multimedia health risk assessments (HRAs) are influenced by both population variability and uncertainty in data and modeling assumptions arising from emission estimation, atmospheric dispersion modeling, multimedia transport modeling, and exposure assessment approaches [1,2,3,4]. Municipal solid waste incinerators (MSWIs) are important sources of polychlorinated dibenzo-p-dioxins and dibenzofurans (PCDD/Fs), whose multimedia exposure risks continue to raise concern. Conventional deterministic assessments commonly rely on population-averaged exposure assumptions, which can mask inter-individual variability and thereby underestimate risk while also obscuring uncertainty associated with exposure characterization and toxicity dosimetry, particularly for susceptible early-life subpopulations.

PCDD/Fs are persistent organic pollutants with long biological half-lives and are classified as carcinogenic to humans (Group 1) by the International Agency for Research on Cancer [5]. Experimental and epidemiological evidence indicates that developmental exposures may result in higher lifetime cancer risk than comparable adult exposures [6,7], supporting the plausibility and relevance of early-life susceptibility.

Additional uncertainty arises from the inhalation cancer potency characterization for dioxin. A commonly cited inhalation unit risk (IUR) for TCDD/Fs is 38 (μg/m^3^)^−1^, as reported by the California Office of Environmental Health Hazard Assessment [6]. However, this value was derived from the oral slope factor (SF_oral_) through route-to-route extrapolation rather than from direct inhalation-specific cancer evidence. Moreover, the SF_oral_ itself was derived under default adult exposure assumptions and may not adequately reflect differential susceptibility across life stages. Accordingly, the U.S. Environmental Protection Agency (U.S. EPA) recommends the use of age-dependent adjustment factors (ADAFs) to account for increased carcinogenic susceptibility during early life stages [8,9], thereby reducing uncertainty associated with applying adult-based toxicity parameters to early-life exposures.

Uncertainty in dioxin risk assessment also arises from exposure characterization. Previous MSWI multimedia exposure studies in northern Taiwan identified food ingestion, particularly above-ground vegetables, eggs, poultry, and fruit, as the dominant contributor to overall exposure variance [1], indicating that age-specific intake per body weight (CR/BW) is a major driver of exposure uncertainty. Probabilistic approaches such as Monte Carlo simulation (MCS) are then applied to explicitly quantify population variability and propagate uncertainty through exposure parameter distributions [10,11].

Recent international studies have continued to highlight the importance of assessing health risks associated with MSWI-related PCDD/F emissions and residential exposure near incineration facilities [10,12,13]. In addition, recent research has confirmed that PCDD/Fs can persist in the environment and biomagnify through food chains, making dietary exposure an important pathway [7,14]. These findings support the use of a site-specific multimedia framework that integrates atmospheric dispersion, food-chain transfer, and probabilistic analysis.

The objective of this study is to develop a probabilistic framework to reduce uncertainty and quantify variability in multimedia carcinogenic risk assessment for susceptible populations, as illustrated by a case study of MSWI emissions in Taiwan. Specifically, this study aims to (1) evaluate the extent to which ADAF-based adjustment reduces uncertainty in risk estimates for susceptible groups; (2) quantify inter-individual variability in multimedia risk estimation using a bivariate MCS approach; and (3) systematically examine uncertainty associated with gas–particle partitioning, ADAF adjustment, and extrapolation of the inhalation slope factor from the oral slope factor.

## 2. Methods

### 2.1. Study Site and Emission Rates

**Figure 1:**
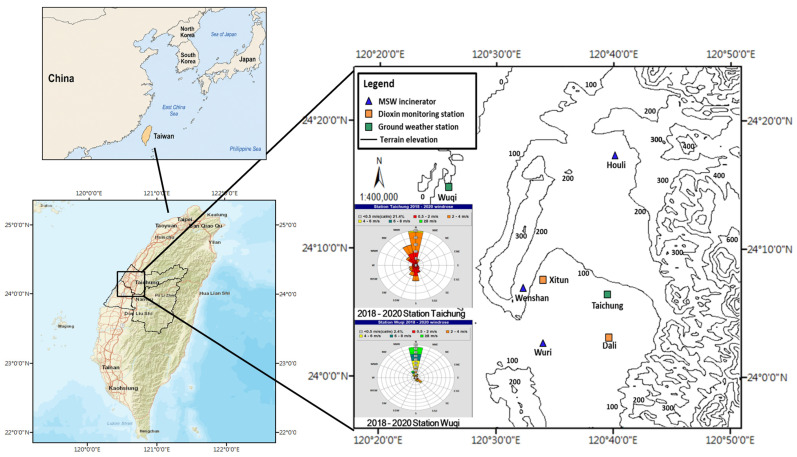
Map of the 40 km × 40 km study area, including locations of three MSW incinerators (Houli, Wenshan, and Wuri), two dioxin monitoring stations (Xitun and Dali), two surface weather stations (Taichung and Wuqi), and wind roses. The inset maps show the location of Taiwan in East Asia and the position of the central Taiwan study area within Taiwan.

The study site is located in central Taiwan, the second-largest metropolitan region in the country, with a population of approximately 3.0 million. Due to the high population density (≥750 people/km^2^), the urban mode was applied in AERMOD simulations. Figure 1 presents a map of the 40 km × 40 km study area. There are three large MSWIs (Houli, Wenshan, and Wuri), two dioxin monitoring stations (Xitun and Dali), and two meteorological stations (Taichung and Wuqi) in the area. The terrain near each incinerator varies in elevation—400 m east of Houli, 300 m north of Wenshan, and 200 m southwest of Wuri—all of which exceed the incinerator stack heights, creating complex terrain conditions that may influence pollutant dispersion.

Meteorological data from the Taichung and Wuqi stations were collected for the period 2018–2020. Figure 1 also displays wind roses indicating prevailing wind patterns, predominantly coming from the north (24.9% and 42.0% at Taichung and Wuqi, respectively), followed by north–northwest (15.2% and 9.99%). The Taichung station, situated in a southern urban area, recorded prevailing wind speeds of 0.5–2 m/s (52.2%), followed by 2–4 m/s (27.6%). Meanwhile, the Wuqi station, near the western coast, experienced wind speeds primarily in the range of 2–4 m/s (32.5%), followed by 4–6 m/s (25.0%).

Table 1 summarizes the design capacities of the three incinerators: Houli (450 metric tons/day × 2 units), Wenshan (300 metric tons/day × 3 units), and Wuri (450 metric tons/day × 2 units). Collectively, these facilities process 2700 metric tons of MSW daily and generate 420 megawatts of power [15]. Quarterly PCDD/Fs emission data from 2018 to 2020 were obtained from the Taiwan EPA [16]. Emissions were analyzed for 17 congeners with internationally defined toxicity equivalency factors (I-TEF) [17]. The average emission factors (ng-TEQ/mt) were estimated as 34.5 for Houli (*n* = 12), 102.4 for Wenshan (*n* = 12), and 34.6 for Wuri (*n* = 6). These values fall within the range reported in the literature (13.8–660 ng-TEQ/mt) [2,3,4], indicating that dioxin emissions from the three facilities were relatively well controlled.

Under design capacity operating conditions, the estimated PCDD/F emission rates for the three incinerators were 3.59 × 10^−10^ g-TEQ/s for Houli, 1.07 × 10^−9^ g-TEQ/s for Wenshan, and 3.60 × 10^−10^ g-TEQ/s for Wuri. Based on the gas-phase (<0.3 μm) and particulate-phase (≥0.3 μm) partitioning data from large-scale incineration studies in Taiwan [18], total emissions were partitioned into 90% gaseous and 10% particulate fractions, as shown in Table 1.

### 2.2. AERMOD Modeling

AERMOD was designated as the preferred air dispersion model by the U.S. EPA in 2005, replacing ISCST3 (Industry Source Complex Short-Term 3) [19]. In 2023, Taiwan also adopted AERMOD as the preferred dispersion model [20]. In this study, AERMOD version 21112 was used for air dispersion modeling, and ISCST3 was used for model comparison. Both AERMOD and ISCST3 are steady-state Gaussian plume models. AERMOD utilizes AERMET and AERMAP for preprocessing meteorological and terrain data, respectively, before conducting dispersion simulations.

AERMET requires meteorological inputs, including wind speed, wind direction, temperature, atmospheric pressure, rainfall, cloud cover, and three surface characteristics (surface roughness, Bowen ratio, and albedo) based on local land use and seasonal conditions [21]. Taiwan’s land use is categorized into 24 types, divided into 439 meteorological blocks (each measuring 10 km × 10 km). The simulation period spanned from 2018 to 2020, with meteorological blocks selected based on the central location of the incinerator. Ground meteorological data were obtained from the Wuqi station for Houli and Wenshan and from the Taichung station for Wuri, while upper-air meteorological data were sourced from the Magong station [22].

AERMAP generates terrain data of elevations and hill-scale heights at each 100 m × 100 m grid across the study site. AERMOD then estimates the dividing streamline height at each grid to determine whether plumes disperse around or over complex terrain. If the slope of the terrain surrounding a receptor point is ≥10%, the maximum nearby hill height is used as the receptor’s hill-scale height; otherwise, the initial terrain height is retained [23]. Since each incinerator falls within a different meteorological block, separate simulations were conducted for each facility. The simulation values at the same grid point were then aggregated across the three incinerators, covering a total of 139,287 grids. The same meteorological and terrain datasets were also used in ISCST3 to compare its simulation results with those of AERMOD, thereby allowing model performance to be evaluated under consistent input conditions and reducing uncertainty associated with inter-model comparison.

### 2.3. MEPAS Modeling and Exposure Estimation

Multimedia transport and exposure to PCDD/Fs were evaluated using the MEPAS model version 5.0 [24], which converts AERMOD-simulated ground-level concentrations and deposition rates into medium-specific values for seven exposure pathways: inhalation (air and dust) and ingestion (soil, leafy vegetables, other vegetables, meat, and dairy products). Exposure was quantified separately for inhalation and ingestion pathways in accordance with the U.S. EPA Exposure Factors Handbook (EFH) framework [25].

For inhalation exposure, the average daily dose via inhalation (ADD_inh_, mg/kg/day) was estimated as follows:

(1) $${ADD}_{inh}={EC}_{air}\times (\frac{IR}{BW})\times \frac{ET}{AT}$$

where EC_air_ is the modeled excess concentration of PCDD/Fs in air (mg/m^3^) simulated from MEPAS, IR/BW is the inhalation rate per body weight (m^3^/day/kg bw), ET is the exposure time for the assessed populations (70 years for the general population from 1 to 70 years and 7 years for school children from 7 to 13 years), and AT is the averaging time for carcinogenic effects (70 years). The BW values were obtained from the Taiwan Health Promotion Administration [26], while the IR values were estimated using Layton’s equation [27,28], based on caloric intake data from THPA [26].

For ingestion exposure, the average daily dose via ingestion (ADD_oral_, mg/kg/day) was calculated for each medium as follows:

(2) $${ADD}_{oral}={EC}_{mdm}\times (\frac{CR}{BW})\times \frac{ET}{AT}$$

where EC_mdm_ is the modeled excess concentration of PCDD/Fs in each ingestion medium (mg/kg), and CR/BW is the consumption rate per body weight (g/day/kg bw) for each ingestion medium, estimated from the 2005–2012 Nutrition and Health Survey in Taiwan (NAHSIT) [29]. This formulation directly incorporates BW-normalized intake and is consistent with the U.S. EPA Exposure Factors Handbook for representing population variability in dietary exposure [25].

Table 2 presents the exposure factors for various media. The assessed populations comprised the general population (1 to 70 years) and school children (7 to 13 years). The general population was further stratified into adults (16 to 70 years) and two susceptible subgroups: toddlers (1 to <2 years) and children (2 to <16 years). These age-specific exposure parameters were incorporated to better capture inter-individual variability and improve the reliability of multimedia risk estimates for susceptible populations.

### 2.4. Cancer Risk Estimation Approaches

For comparison, excess cancer risk (ECR) was estimated using three assessment approaches: (1) a deterministic method without toxicity adjustment; (2) a deterministic method with toxicity adjustment; and (3) a probabilistic method with toxicity adjustment. The first represents the conventional framework, the second shows the effect of age-dependent toxicity adjustment, and the third further incorporates inter-individual variability in body weight-normalized inhalation and ingestion exposure through probabilistic simulation.

ECR was estimated separately for inhalation and oral exposure as the product of the applicable slope factor (SF) for PCDD/Fs and the corresponding ADD. To account for increased carcinogenic susceptibility during early life, ADAFs recommended by the U.S. EPA were applied: 10 for toddlers aged 1 to <2 years, 3 for children aged 2 to <16 years, and 1 for adults aged ≥16 years [8,9]. The inhalation and oral ECR values were calculated using Equations (3) and (4), respectively.

(3) $${ECR}_{inh}=\left({SF}_{inh}\right)\times ({ADD}_{inh})\times \left(ADAF\right)$$

(4) $${ECR}_{oral}=({SF}_{oral})\times ({ADD}_{oral})\times (ADAF)$$

where SF_oral_ is the oral SF for PCDD/Fs, derived from a 104-week feeding study in rats and mice [6,30], and SF_inh_ is the inhalation SF extrapolated from SF_oral_. The same value, 1.3 × 10^5^ (mg TEQ/kg bw/day)^−1^, was applied to SF_inh_ and SF_oral_. ADD_inh_ and ADD_oral_ denote the ADD for inhalation and oral exposure, respectively.

The general population was further stratified into adults (16 to 70 years) and two susceptible subgroups: children (2 to <16 years) and toddlers (1 to <2 years). ECR for the general population (ECR_GP_) and school children (ECR_SC_) (7 to 13 years) was estimated from age-specific ADDs using Equations (1) and (2), together with the corresponding SF_inh_ and SF_oral_ and applicable ADAF, as shown in Equations (5) and (6), respectively.

ECR_GP_ = SF × ADD_1 to <2_ × 10 + SF × ADD_2 to <16_ × 3 + SF × ADD_16 to 70_ × 1 (5)

ECR_SC_ = SF × ADD_7 to 13_ × 3 (6)

The total cancer risk (ECR_total_) for each population was then obtained by summing inhalation and ingestion risks across all relevant exposure media:

(7) $${ECR}_{total}={\sum ECR}_{inh}{+\sum ECR}_{oral}$$

A bivariate MCS was then performed at the P95 receptor grid identified from the conventional population-averaged AERMOD-MEPAS assessment. In this study, “bivariate” refers to the simultaneous use of two individual-level exposure variables: IR/BW for inhalation exposure and CR/BW for ingestion exposure. For each individual, IR/BW and CR/BW were used to calculate pathway-specific ADD values and corresponding cancer risks. The stochastic variables included individual-level IR/BW and pathway-specific CR/BW values for leafy vegetables, other vegetables, meat, milk, and soil ingestion. The distributions and key parameters of these stochastic variables, including summary statistics and fitted distribution types, are provided in the Supplementary Materials. The resulting individual risks (*n* = 7058 for the general population and *n* = 1879 for school children) were fitted to the best-fitting distributions, which were selected based on goodness-of-fit statistics generated by Crystal Ball before performing 10,000 Monte Carlo iterations to characterize the distribution of cancer risk. Crystal Ball was used to perform the MCS, distribution fitting, and visualization of the probability density functions, while the R-based computational program (Supplementary Materials) reproduces the complete computational workflow.

## 3. Results and Discussion

### 3.1. Framework Overview and Summary of Risk Estimates

**Figure 2:**
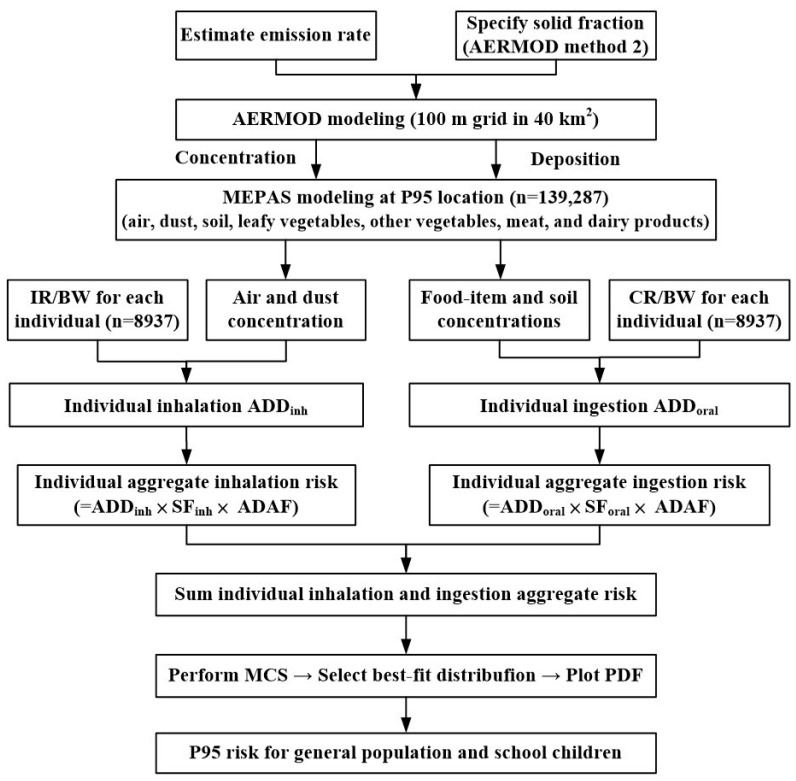
The proposed bivariate probabilistic framework for multimedia dioxin risk assessment, integrating emission estimation, phase partitioning, AERMOD, MEPAS, age-specific exposure assessment, ADAF-adjusted slope factor (SF), and Monte Carlo simulation (MCS).

Figure 2 presents the bivariate probabilistic framework developed to reduce uncertainty in multimedia dioxin risk assessment. The framework integrates design capacity emission estimation and gas–particle partitioning with ISCST3 or AERMOD simulation over a 40 × 40 km area at 100 m resolution, followed by MEPAS modeling to derive concentrations in air, dust, soil, leafy vegetables, other vegetables, meat, and dairy products across 139,287 receptor grids.

These outputs were then used to estimate pathway-specific ADDs for 8937 individuals based on individual IR/BW and CR/BW. SF_inh_ and SF_oral_ were adjusted using ADAF to reflect differential carcinogenic susceptibility across life stages. Finally, inhalation and ingestion risks were aggregated across pathways, and MCS with 10,000 iterations was used to characterize the distributions of individual cancer risks for the general population and school children.

**Figure 3:**
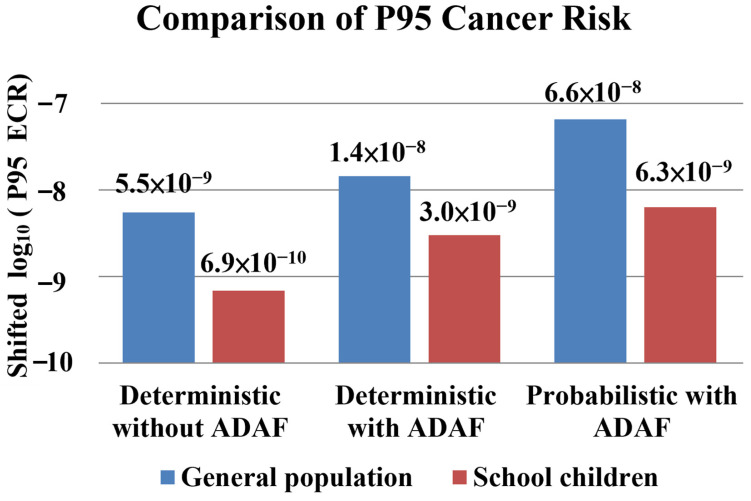
Comparison of P95 cancer risk estimates for the general population and school children under deterministic and probabilistic approaches with and without ADAF adjustment. The stepwise increase in risk illustrates the effects of toxicity adjustment in reducing uncertainty and probabilistic simulation in quantifying inter-individual variability.

Figure 3 compares the P95 cancer risk estimates for the general population and school children under three assessment approaches: deterministic without ADAF adjustment, deterministic with ADAF adjustment, and probabilistic with ADAF adjustment. The stepwise increase in risk across these approaches illustrates how the proposed framework reduces uncertainty and quantifies variability in multimedia dioxin risk assessment. For the general population, the P95 risks increased from 5.5 × 10^−9^ under the conventional deterministic approach to 1.4 × 10^−8^ (a 2.54-fold increase) after ADAF adjustment and further to 6.6 × 10^−8^ (an additional 4.71-fold increase) when inter-individual variability in IR/BW and CR/BW was incorporated through probabilistic simulation. For school children, the corresponding P95 risks increased from 6.9 × 10^−10^ to 3.0 × 10^−9^ (a 4.35-fold increase) and then to 6.3 × 10^−9^ (an additional 2.1-fold increase).

These results indicate that the conventional population-average approach may underestimate cancer risk by not accounting for age-dependent susceptibility and variability in BW-normalized exposure. Thus, ADAF adjustment reduced uncertainty associated with life-stage extrapolation, whereas the probabilistic approach further quantified variability in inhalation and dietary exposure. The respective contributions of these methodological refinements are discussed in the following sections.

### 3.2. Deterministic Risk Mapping

**Figure 4:**
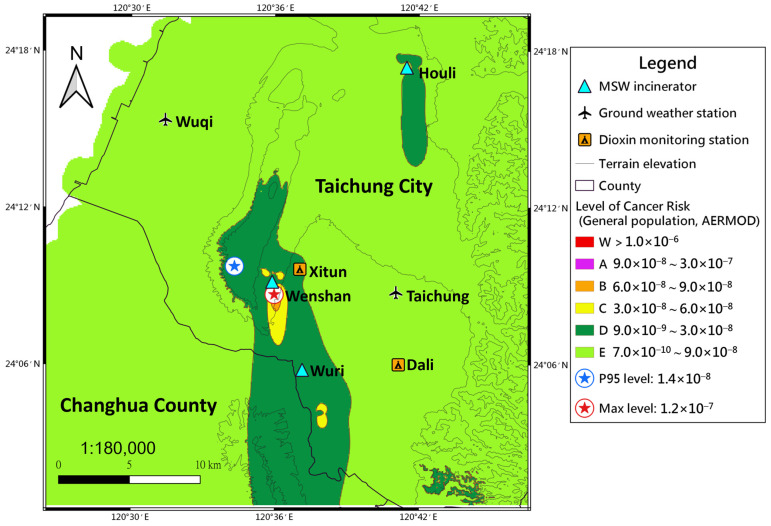
Five-grade deterministic cancer risk map for the general population in central Taiwan generated by the AERMOD + MEPAS simulation with ADAF adjustment for early-life susceptibility, showing the locations of the P95 and maximum risk receptors across all grids (*n* = 139,287) within the 40 km × 40 km study area.

Figure 4 presents the five-grade population-average cancer risk map generated by the AERMOD + MEPAS simulation for the general population with ADAF adjustment for early-life susceptibility. Risk values were classified into five grades (A to E), with values exceeding 10^−6^ marked in red as grade “W” (warning). The map also identifies key landmarks, including three incinerators, surface meteorological stations, dioxin monitoring stations, terrain contour lines, county boundaries, and the locations of P95 and maximum risk receptors.

The AERMOD + MEPAS results exhibited a smoother and continuous dispersion pattern across the complex terrain study area. For the general population, the P95 deterministic risk across all 139,287 grids within the 40 km × 40 km study area was 1.4 × 10^−8^ (blue star), whereas the maximum risk was 1.2 × 10^−7^ (red star). Both high-risk locations were concentrated near the Wenshan plant, indicating that this facility dominated the spatial risk pattern. This distribution is consistent with the higher dioxin emissions from the Wenshan plant (Table 1) and its location within a narrow valley, where terrain confinement likely restricted plume dispersion and increased ground-level concentrations. In addition, more than 75% of the total cancer risk was attributable to food ingestion pathways, consistent with previous multimedia exposure studies [1,3,4].

Compared with ISCST3, the AERMOD-based simulation produced a less fragmented and more physically realistic risk pattern under complex terrain conditions. In the illustrated case study, the deterministic P95 cancer risk estimated using AERMOD + MEPAS was 1.4 × 10^−8^, which was approximately one-third of the corresponding ISCST3 + MEPAS estimate (4.4 × 10^−8^). This difference reflects AERMOD’s ability to account for vertical overflow and horizontal plume splitting, whereas ISCST3 tends to overpredict concentrations at plume–terrain impact points because of its more limited treatment of complex topography [31].

The deterministic simulation also showed that the P95 risks for school children were 12.5% for the general population without ADAF adjustment and 21.4% with ADAF adjustment, mainly because school children have a shorter exposure duration over a 70-year lifetime. However, subsequent ADAF adjustment and probabilistic simulation demonstrated that risks for susceptible populations can increase substantially when early-life susceptibility and inter-individual variability are explicitly considered. These effects are further examined in the following sections.

### 3.3. Monte Carlo Simulation

**Figure 5:**
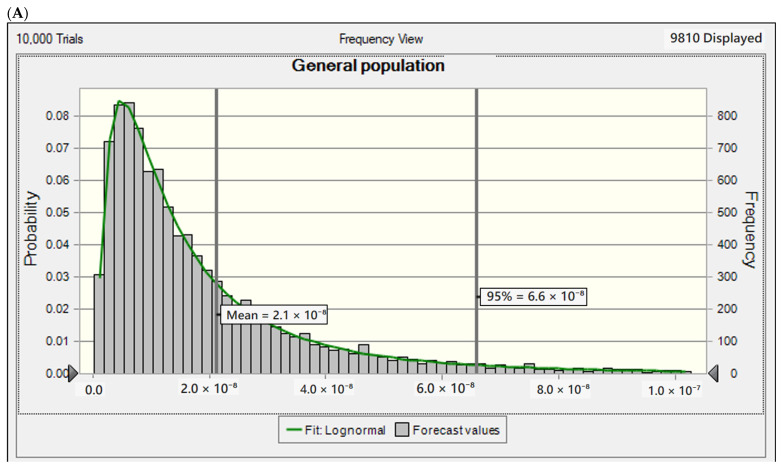

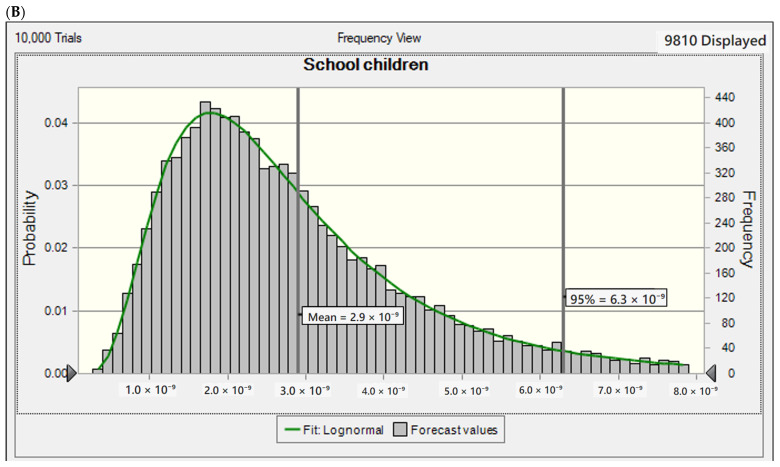
Best-fitting lognormal distributions of individual excess cancer risk, with mean and P95 risks indicated for the general population (**A**) and school children (**B**) based on MCS at the P95 receptor grid identified from the deterministic risk map of Figure 4. The probability density functions were fitted and visualized using Crystal Ball.

Figure 5 shows the best-fitting lognormal distributions of individual excess cancer risk. The distributions were derived from 10,000 Monte Carlo simulations performed at the P95 receptor grid identified from the conventional deterministic assessment after ADAF adjustment for early-life susceptibility. For the general population (*n* = 7058), the mean and P95 risks were 2.1 × 10^−8^ and 6.6 × 10^−8^, respectively, whereas for school children (*n* = 1879), the corresponding values were 2.9 × 10^−9^ and 6.3 × 10^−9^. All probabilistic risk estimates remained below the benchmark level of 10^−6^. Therefore, these results should not be interpreted as indicating unacceptable risk. Rather, their main significance is methodological and decision-supportive: the proposed framework shows how age-dependent susceptibility and inter-individual variability can increase upper-tail risk estimates while still remaining within an acceptable risk range. This provides a more transparent and balanced basis for risk communication, policy interpretation, and prioritization of susceptible-population assessment.

The probabilistic P95 risks were 4.6 times higher for the general population and 2.1 times higher for school children than the corresponding deterministic estimates. These differences reflect the influence of individual-level variability in IR/BW and CR/BW, which is not captured by population-average deterministic methods. The results indicate that incorporating individualized exposure metrics, together with toxicity adjustment, improves characterization of upper-tail risk and helps identify the extent to which the conventional deterministic approach may underestimate risk, particularly for susceptible populations.

### 3.4. Uncertainty Analysis

Because uncertainty analysis supports the interpretation of deterministic and probabilistic risk estimates, it was integrated into Section 3. Building on the recent literature summarized in Section 1, the present analysis focused on three uncertainty sources that directly affect the interpretation of the PCDD/F multimedia risk estimates: gas–particle partitioning, ADAF adjustment, and slope factor extrapolation. Recent probabilistic assessments also support the use of MCS for characterizing exposure variability and parameter uncertainty [10,11].

#### 3.4.1. Gas–Particle Partitioning

Gas–particle partitioning is a critical but often overlooked source of uncertainty in the dispersion modeling of PCDD/Fs. In accordance with U.S. EPA AERMOD guidance [32,33], gaseous and particulate fractions were modeled separately because gas deposition and particulate deposition require different input options and deposition parameters, with particulate deposition specified using Method 1 or Method 2. This phase-specific treatment improves the reliability of subsequent dispersion modeling and multimedia risk estimates.

In this study, to reduce uncertainty in particulate deposition, particle-size distribution data reported for large-scale MSWIs [34,35] were adopted, with mass fractions of 87.5% at 1.0 μm, 9.5% at 6.78 μm, and 3.0% at 20 μm. These data were reclassified to a cut size of <1.5 μm, representing 97% of the total particulate phase and satisfying the AERMOD Method 2 requirement that particles <10 μm account for at least 90% of the total phase. Accordingly, these particle-size and mass fraction data were used in particulate deposition simulations in ISCST3 to ensure comparability with AERMOD. Explicit specification of particle sizes and mass fractions reduces uncertainty in dry and wet deposition estimates and thereby improves the reliability of subsequent multimedia transport modeling.

#### 3.4.2. ADAF Adjustment

ADAF adjustment addressed one source of uncertainty in cancer risk estimation by explicitly accounting for the greater carcinogenic sensitivity of early-life exposure. In the illustrated case study, ADAF adjustment increased the toxicity-adjusted deterministic P95 risks by 2.54-fold for the general population and 4.35-fold for school children. The smaller increase for the general population is because this group includes adults aged 16 years or older, for whom the ADAF is 1, and the longer adult exposure duration dominates the lifetime risk estimate. As a result, the influence of ADAF adjustment applied to early-life subgroups is diluted in the overall general population estimate. In contrast, the larger increase in school children (2 to <16 years) reflects both greater biological susceptibility during development and the application of an ADAF of 3. Thus, ADAF adjustment improved the biological relevance of age-specific risk estimates while addressing uncertainty associated with applying adult-based toxicity assumptions to susceptible populations [8,9].

It should be noted that ADAFs are recommended by the U.S. EPA primarily for carcinogens with a mutagenic mode of action involving DNA reactivity, whereas carcinogens acting through other mechanisms generally require chemical-specific evaluation rather than default age-based adjustment. Because dioxin-like compounds, including TCDD/Fs, are generally considered AhR-mediated and non-mutagenic, the applicability of ADAF to dioxin is not straightforward. Nevertheless, given the importance of early-life susceptibility, long biological persistence, and cumulative body burden, ADAF adjustment was applied in this study as a conservative component of the uncertainty analysis rather than as a default requirement for dioxin risk assessment [8,36]. In the probabilistic analysis, ADAF values were incorporated through individual age information. Each individual was assigned the corresponding age-dependent factor according to their age group before calculating the individual cancer risk. Therefore, the influence of age-dependent susceptibility was reflected in the MCS through the age structure of the study population. However, ADAF values were not treated as stochastic variables because they are categorical regulatory adjustment factors rather than empirically derived probability distributions.

#### 3.4.3. Slope Factor Extrapolation

In this study, the SF_oral_ for dioxin was extended to inhalation to provide a unified dose-based cancer assessment across exposure routes. This approach improves internal consistency within the multimedia framework by allowing inhalation and oral risks to be evaluated on the same toxicity basis while incorporating ADAF adjustment and age-specific exposure parameters. However, this route-to-route extrapolation remains a source of uncertainty because it assumes comparable absorption and internal dosimetry between oral and inhalation exposures, which may not be fully equivalent in practice.

This source of uncertainty should be interpreted in the context of available regulatory values. The current U.S. EPA IRIS summary for TCDD/Fs does not provide a finalized quantitative inhalation cancer value, indicating that inhalation-specific cancer potency remains unresolved in that framework [37]. In contrast, the California Office of Environmental Health Hazard Assessment (OEHHA) reports the same dose-based oral slope factor, 1.3 × 10^5^ (mg/kg-day)^−1^, for both oral and inhalation exposure to TCDD/Fs, based on a 104-week feeding study in rats and mice [6,30], and also lists an IUR of 3.8 × 10^1^ (μg/m^3^)^−1^ [6]. Notably, the OEHHA inhalation value was also derived by route-to-route extrapolation rather than from direct inhalation-specific cancer evidence.

Accordingly, the inhalation cancer estimates in this study should be regarded as internally consistent, although slope factor extrapolation remains a source of uncertainty in the proposed risk assessment framework. Nevertheless, the use of the extrapolated SF_inh_ was advantageous for the bivariate MCS because it allowed age-dependent ADD_inh_ to be calculated using Equation (1). Because the inhalation rate (IR) was estimated from age-dependent dietary caloric intake following Layton [27], its explicit inclusion enabled physiological differences across life stages to be reflected in inhalation exposure. In contrast, if inhalation cancer risk had been estimated using the IUR, the IR would not have explicitly appeared in the risk equation, and inter-individual variability in IR/BW could not have been incorporated into the MCS. Although slope factor extrapolation represents an important source of uncertainty, it was not incorporated as a stochastic variable in the MCS because no defensible probability distribution was available for the extrapolated inhalation slope factor. Accordingly, this uncertainty was addressed qualitatively in the uncertainty analysis rather than quantitatively propagated through the MCS. Future studies may incorporate toxicity parameter uncertainty into probabilistic simulations when chemical-specific distributions or confidence limits become available.

## 4. Conclusions and Recommendations

In this study, a bivariate probabilistic framework was developed to address selected uncertainty sources and quantify variability in multimedia dioxin carcinogenic risk assessment for susceptible populations, as illustrated by emissions from MSWIs in Taiwan. The framework integrates design capacity emission estimation, gas–particle partitioning, AERMOD dispersion modeling, MEPAS multimedia transport modeling, age-specific exposure characterization, ADAF-based toxicity adjustment, and bivariate MCS. Deterministic P95 risk increased substantially after ADAF adjustment, indicating that conventional population-average assessment may have underestimated risks for susceptible groups. Further increases under probabilistic analysis demonstrated the importance of inter-individual variability in body weight-normalized inhalation and ingestion exposure. Uncertainty analysis also identified gas–particle partitioning, dispersion modeling, and slope factor extrapolation as important contributors to overall risk uncertainty.

Overall, the proposed framework improved the biological relevance, internal consistency, and transparency of multimedia cancer risk estimates and provides a transferable approach for future multimedia risk assessments of persistent pollutants in susceptible populations. The novelty of this study lies in the integration of site-specific high-end receptor identification, multimedia fate and transport modeling, age-dependent susceptibility adjustment, and individual-level probabilistic exposure characterization within a single framework to address selected uncertainty sources and quantify variability. Although PCDD/F emissions from MSWIs were used as the illustrative case in this study, the framework may also be extended to other persistent, bioaccumulative, or multimedia fate and transport contaminants when compound-specific emission, partitioning, multimedia transfer, toxicity, and exposure data are available.

## Figures and Tables

**Table 1:** Design capacities and PCDD/Fs emission data (2018–2020) of the three MSW incinerators in central Taiwan.

Item	Houli	Wenshan	Wuri
Incinerators and stacks data			
Design treatment capacity (mt/day)	450 × 2	300 × 3	450 × 2
Number of stacks	2	3	2
Stack height (m)	120	120	100
Stack diameter (m)	1.70	1.76	1.65
Dioxin emission data *^a^*			
Average treatment capacity (mt/day)	853.8	627.5	910.2
Average dioxin concentration (ng-TEQ/Nm^3^)	1.36 × 10^−2^	4.02 × 10^−2^	1.25 × 10^−2^
Average exit velocity (m/s)	11.7	15.0	21.5
Average exit flow (Nm^3^/min)	1508	1040	1748
Average emission factor (ng/mt)	34.5	102.4	34.6
Design total emission rate (g/s)	3.59 × 10^−10^	1.07 × 10^−9^	3.60 × 10^−10^
Design gaseous phase emission rate (g/s)	3.23 × 10^−10^	9.60 × 10^−10^	3.24 × 10^−10^
Design particulate phase emission rate (g/s)	3.59 × 10^−11^	1.07 × 10^−10^	3.60 × 10^−11^

*^a^* Number of dioxin data: Houli (*n* = 12), Wenshan (*n* = 12), Wuri (*n* = 6).

**Table 2:** Exposure factors for various media in the general population and susceptible groups.

Exposure Factors	General Population (1–70 Years)			School Children (7–13)	Units	Data Source
	Toddler (1–<2)	Children (2–<16)	Adult (16–70)			
Body weight	12.6	35.1	64.8	39.5	kg	[26]
Exposure time	1	14	55	7	year	
Age lower bound	1	2	16	7	year	
Age upper bound	1	15	70	13	year	
Inhalation rate	6.8	11.4	13.9	12.6	m^3^/day	[26]
Consumption rate						
Leafy vegetables	1.81	2.96	2.97	3.03	g/day/kg bw	[29]
Other vegetables	16.84	15.87	11.16	14.50	g/day/kg bw	[29]
Meat	1.87	4.86	2.85	4.87	g/day/kg bw	[29]
Milk	16.90	4.94	0.96	3.47	g/day/kg bw	[29]
Soil (pica)	0.05	0.05	0.02	0.05	g/day	[25]

## Data Availability

The data presented in this study are available on request from the corresponding author.

## Supplementary Materials

The following supporting information can be downloaded at: https://www.mdpi.com/article/10.3390/toxics14070571/s1, File S1: R program for reproducing the MCS workflow of probabilistic ECR distributions. The MCS program, implemented in R, incorporated individual-level variability in age-specific inhalation and ingestion exposures (IR/BW and CR/BW) and applied ADAF adjustment, generating 10,000 probabilistic ECR distributions across two study populations.
